# DNA- and telomere-damage does not limit lifespan: evidence from rapamycin

**DOI:** 10.18632/aging.202674

**Published:** 2021-02-12

**Authors:** Mikhail V. Blagosklonny

**Affiliations:** 1 Roswell Park Cancer Institute, Buffalo, NY 14263, USA

**Keywords:** quasi-programmed aging, hyperfunction theory, antagonistic pleiotropy, natural selection, mTOR

## Abstract

Failure of rapamycin to extend lifespan in DNA repair mutant and telomerase-knockout mice, while extending lifespan in normal mice, indicates that neither DNA damage nor telomere shortening limits normal lifespan or causes normal aging.

## INTRODUCTION

As a provocative title has recently announced, “rapamycin fails to extend lifespan in DNA repair-deficient mice” [1]. The word “fails” implies bad news. Rapamycin tried but failed. Yet, it is expected that the anti-aging drug rapamycin should not restore lifespan of short-lived mice that fail to grow and die young from causes other than normal aging [2]. In such growth-retarded mice, rapamycin, an inhibitor of cell growth, further retards weight gain.

Similarly, rapamycin does not extend but even slightly shortens lifespan in telomerase-deficient mice, which die young from poor growth and intestinal atrophy caused by telomere shortening [3]. (As we will discuss, this is predictable by hyperfunction theory.) While shortening lifespan by 18% in unnatural telomerase-deficient mice, in the same study in natural mice, rapamycin increased lifespan by 39% and healthspan by 58% (measured as tumor-free survival) [3]. In dozens of independent studies, rapamycin has not failed to extend lifespan in normal mice [4]. However, while extending lifespan in normal mice, rapamycin may fail to save animals dying young from cellular growth retardation. But something important should not be overlooked. The failure of rapamycin to extend lifespan in these short-lived mice, dying from DNA damage, rules out the damage theory of aging. To understand this point, we must first discuss what limits animal lifespan.

### Quasi-programmed (hyperfunctional) aging

In proliferating cells, growth-promoting pathways such as mTOR (Target of Rapamycin) and MAPK drive cellular growth, which is balanced by cell division. When the cell cycle is arrested, however, growth-promoting pathways drive cellular senescence, which is a continuation of cellular growth in the absence of cell division [5]. During geroconversion to senescence, cells become hypertrophic and hyperfunctional. One example of hyper-function is SASP or Senescence-Associated Secretory Phenotype [6]. Rapamycin can cause reversible cycle arrest but suppresses geroconversion, thus ensuring quiescence instead of senescence. (Note: Rapamycin does not prevent cell cycle arrest, it only prevents geroconversion that makes this arrest permanent [7]. This point is often miscited by others). Rapamycin slows down both growth and geroconversion, figuratively slowing down time [8]. Like cellular senescence is a continuation of growth, organismal aging is a continuation of growth too [9].

According to hyperfunction theory, aging is quasi-programmed, a continuation of developmental growth programs, driven in part by hyper-functional signaling pathways including the mTOR pathway [9]. Hyperfunction is an excessive normal function later in life. It’s not necessarily an increase of function; it may even be insufficient decrease of function. For example, protein synthesis is decreased in *C elegans* but is still too high: its further inhibition extends lifespan [10, 11].

Hyperfunction leads to age-related diseases, secondary organ damage and loss of function. For example, cellular hyperfunctions result in hypertension, culminating in stroke and damage of the brain. Aging is a sum of all age-related diseases [12, 13]. This theory was discussed in detail [9, 14–20] and has gained experimental support [11, 16, 21–26]. I will not discuss it here, just to mention that accumulation of molecular damage is not a driving force of development and therefore of aging. It is hyperfunctional signaling pathways such as mTOR (one of many) that drive both growth and aging, causing age-related diseases that in turn damage organs, leading to secondary loss of function.

**Figure 1:**
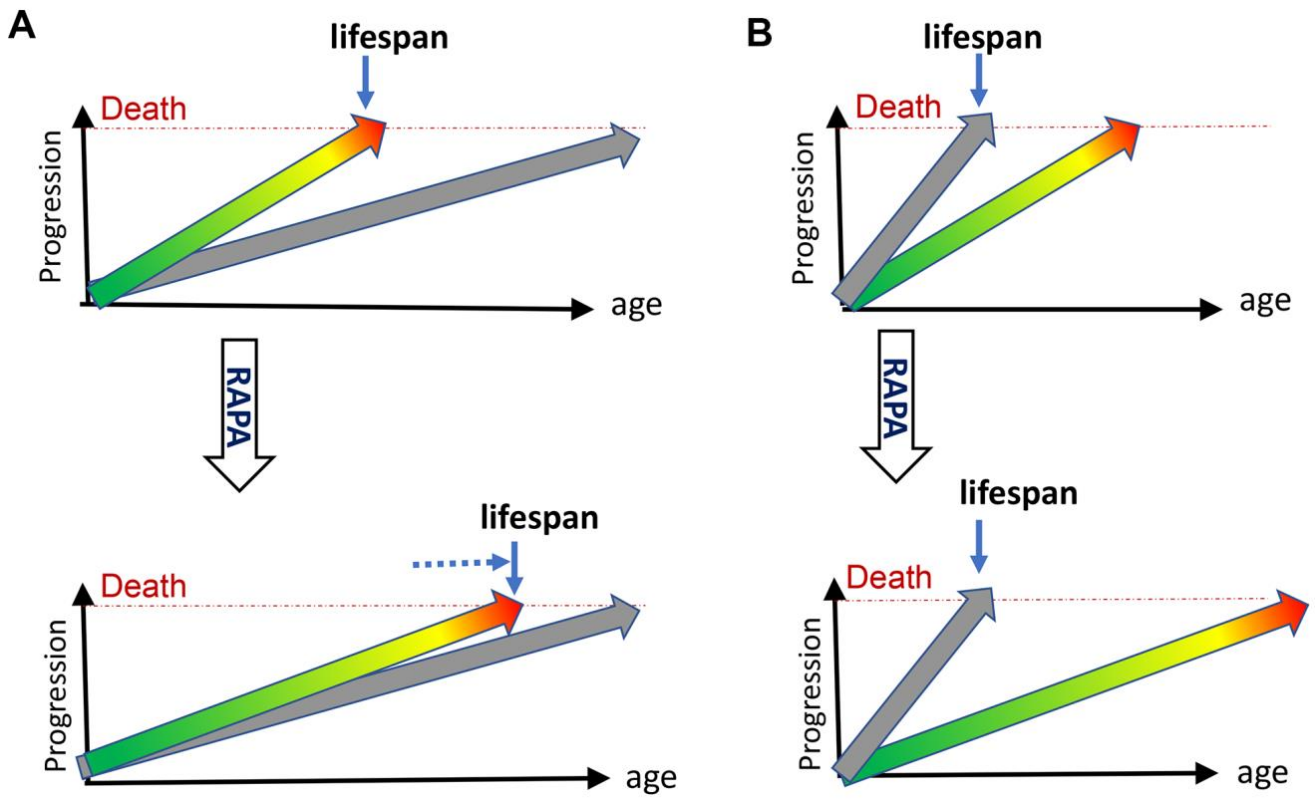
**Rapamycin extends lifespan in natural but not progeroid mice.** (**A**) Natural mice. Hyperfunctional aging (green/yellow/red arrow) progresses from development (green) to diseases (red), reaching death threshold and limiting lifespan. Accumulation of molecular damage (gray arrow) is slow and does not reach death threshold in animal lifetime. It would take longer to die from molecular damage. Treatment with rapamycin (RAPA) extends lifespan by slowing down mTOR-driven aging (**B**) Progeroid, telomerase- or DNA-repair-deficient mice. Accumulation of molecular damage (gray arrow) is artificially accelerated to become life-limiting. Treatment with rapamycin (RAPA) cannot extend lifespan.

Although molecular damage accumulates, this accumulation is not life-limiting because quasi-programmed aging terminates life first (Figure 1A). Quasi-programmed (hyperfunctional) aging is life-limiting, because it is favored by natural selection. Natural selection favors robust development and fitness early in life at the cost of aging. For example, growth hormone receptor-deficient mice (GHR-KO mice), with decreased mTORC1 activity, live longer but are small and weak early in life [27, 28]. In such mice mTORC1-driven aging is inhibited and mice live longer but would not survive in the wild and therefore do not exist in nature. As another example, knockout of PI3K, an activator of mTOR pathways, extends lifespan 10-fold in *C. elegans* [29]. The mutant worm undergoes prolonged developmental arrest, which would be lethal in the wild [29]. Therefore, natural selection favors hyperfunctional mTOR that is optimal for development but drives age-related diseases later in life.

According to damage theories, aging is functional decline caused by molecular damage. According to hyperfunction theory, quasi-programmed aging is not functional decline but a hyperfunction: cellular and systemic functions are higher than optimal for longevity. They are optimal for early life fitness and in part (only in part) mTOR-dependent.

In both molecular damage and hyperfunction theories, aging exists because late-life is shadowed from natural selection. But quasi-programmed aging is not simply shadowed from, it is promoted by natural selection, because accelerated aging is hardwired with fitness early in life. By selecting for fitness, nature indirectly selects for accelerated aging. This makes quasi-programmed aging life-limiting. One of predictions of hyperfunction theory is that rapamycin must extend lifespan in animals [9]. This prediction has been confirmed. In dozens of studies, rapamycin prolongs lifespan and healthspan in mice [3, 30–65]. Rapamycin extends lifespan in *C elegans* [66] and *Drosophila* [67–69]. Furthermore, rapamycin even extends life of the simplest animal, *Hydra*, which is thought to be immortal. Depending on conditions, *Hydra* can be either immortal or undergo aging. Rapamycin slows aging, stem cell exhaustion and extends life span in *Hydra* [70].

mTOR-driven aging is only one component of quasi-programmed (hyperfunction) aging. In addition, MEK/MAPK, NF-kB, p63, HIF-1 and many other signaling pathways are involved, interacting with the mTOR pathway and forming networks. Rapamycin cannot affect all of them. In theory, mTOR-independent quasi-programmed aging can be life-limiting in some conditions and diseases. I suggest that long-lived GHR-KO mice with low mTORC1 activity undergo partially mTORC1-independent quasi-programmed senescence, because rapamycin cannot prolong lifespan in these mice further, while prolonging lifespan in parental normal mice [71]. Discussion of mTOR-independent components of quasi-programmed aging is beyond the focus of this article. Let us return to stochastic accumulation of molecular damage.

### How molecular damage can become life-limiting

Molecular damage can become life-limiting in two ways. First, hyper-functional aging should be eliminated or slowed down, so an organism lives long enough to die from accumulation of molecular damage. In this scenario, accumulation of molecular damage causes post-aging. Such examples are unknown, but it is a very intriguing possibility. Could a PI3K-null worm [29] with 10-fold longer lifespan die from molecular damage?

Second, accumulation of molecular damage can be greatly accelerated artificially by knockout of repair/maintenance enzymes (Figure 1B). Such animals do not exist in nature. But artificially created, they may provide a glimpse of how post-aging may look. Their pathology differs drastically from normal aging, for example, telomere shortening. Second-generation telomerase-deficient mice (G2 *Terc*^−/−^) with critically short telomeres fail to grow and die young from unfamiliar diseases such as intestinal atrophy due to failure of cell proliferation [3]. When telomeres reach critical length, it can cause DNA-damage response, leading to aplastic anemia, organ fibrosis, atrophy of the small intestine and the spleen, skin and hair lesions. In humans, diseases of short telomeres cause death from bone marrow failure and pulmonary fibrosis [72]. This does not resemble normal aging.

In humans, mice and *C. elegans*, telomere shortening is not life-limiting [73–75]. In mice lacking telomerase, even accelerated telomere shortening is still not life-limiting in the first generation [76]. It took several generations to achieve critically short telomeres, leading to syndromes strikingly different from normal aging. In humans, telomere length does not reach telomere threshold during life time [75, 77, 78]. Normal telomere shortening would cause telomere-driven pathologies, but normal animals do not live long enough to reach this threshold. Rapamycin prolongs life in normal mice, proving that telomere length does not constrain normal lifespan [3]. When artificially shortened, then telomeres become life-limiting and rapamycin cannot extend lifespan anymore [3].

*Ercc1*^∆/−^ mutant mice are defective in DNA repair, such as transcription-coupled repair, global-genome nucleotide excision and crosslink repair [1, 2]. Therefore, multiple types of DNA damages accumulate. This leads to decreased cell proliferation, arrested development, poor growth, abnormal liver nuclei of liver and kidney, absence of subcutaneous fat, ferritin deposition, kidney malfunction and early death [2]. Unlike natural mice, short-lived *Ercc1*^∆/−^ mice do not develop tumors, probably because they do not live long enough to suffer typical age-related diseases [1, 2]. In such mice, dying from molecular damage, rapamycin fails to extend lifespan [1].

## CONCLUSIONS

Here I discussed new evidence that normal aging is not caused by accumulation of molecular damage or telomere shortening: while extending normal lifespan in mice, rapamycin failed to do so in mice dying from molecular damage (Figure 1).

Previously, several lines of evidence suggested that molecular damage does not cause normal aging. Their detailed discussion is beyond the focus of this article, so I will just mention some of them, without referencing them (I will reference these points in forthcoming review “When longevity drugs do not increase longevity: Unifying development-driven and damage-induced theories of aging”, In press). First, overexpression of enzymes that decrease damage does not extend lifespan in most studies. Similarly, antioxidants do not extend lifespan in animals and may increase mortality in humans. Furthermore, even data that support damage theory can be explained by other mechanisms. For example, N-Acetyl-L-Cysteine, a commonly used anti-oxidant, can inhibit mTOR. Second, according to calculations, molecular damage, especially mtDNA mutations and telomere shortening, cannot reach deadly threshold during animal lifetime. Third, genetic knockout of signaling pathways can extend lifespan without affecting molecular damage. Similarly, pharmacological interventions can extend life without affecting damage accumulation. Forth, dramatic intra- and inter-species differences in lifespan poorly correlate with the rate of molecular damage. Fifth, nuclear transfer and nuclear reprogramming both rule out DNA damage as a cause of aging. Following adult somatic cell nuclear transfer, cloned animals are healthy and have normal lifespan. Sixth, low levels of molecular damage may increase longevity. This phenomenon is known as hormesis. Regardless of mechanistic explanations, this indicates that molecular damage is not-life-limiting even when moderately increased. Finally, rapamycin increases lifespan in all normal animals tested, indicating that mTORC1-dependent quasi-program is life-limiting. The list can go on and on. Once again, damage accumulates and must cause death eventually, but quasi-programmed (hyperfunctional) aging terminates life first. Molecular damage can become life-limiting, when artificially accelerated or, potentially, when quasi-programmed aging is decelerated. Then interventions to repair molecular damage may increase life further.
